# Identification of immune-related genes and integrated analysis of immune-cell infiltration in melanoma

**DOI:** 10.18632/aging.205427

**Published:** 2024-01-11

**Authors:** Zhenghao He, Manli Chen, Zhijun Luo

**Affiliations:** 1 Department of Plastic Surgery, Zhongshan City People's Hospital, Zhongshan 528403, Guangdong, China

**Keywords:** melanoma, immune infiltration, prognostic model

## Abstract

Objective: This study was conducted to screen out immune-related genes in connection with the prognosis of melanoma, construct a prognosis model and explore the relevant mechanisms.

Methods and materials: 1973 genes associated with immune system were derived from the Immport database, and RNA-seq data of melanoma and information of patients were searched from the Xena database. Cox univariate analysis, Lasso analysis and Cox multivariate analysis were used to screen out six genes to construct the model. Then the risk scores were estimated for patients based on our constructed prognosis model. Estimate was used to affirm that the model was about immune infiltration, and CIBERSORT was used to screen out immune cells associated with prognosis. TIDE was applied to predict the efficacy of immunotherapy. Finally, GSE65904 and GSE19234 were used to confirm the effectiveness of the model.

Results: ADCYAP1R1, GPI, NTS might cause poor prognosis while IFITM1, KIR2DL4, LIF were more likely conductive to prognosis of melanoma patients and a model of prognosis was constructed on the basis of these six genes. The effectiveness of the model has been proven by the ROC curve, and the miRNAs targeting the screened genes were found out, suggesting that the immune system might impact on the prognosis of melanoma by T cell CD8^+^, T cell CD4^+^ memory and NK cells.

Conclusions: In this study, the screened six genes were associated with the prognosis of melanoma, which was conductive to clinical prognostic prediction and individualized treatment strategy.

## INTRODUCTION

Melanoma, a prevalent cutaneous malignancy, arises from melanocytes and exhibits a propensity for metastasis to diverse organs via lymphatic pathways [1, 2]. Heightened aggressiveness has been identified as a primary contributor to mortality in skin cancer cases [3, 4]. Middle-aged individuals are predisposed to melanoma, with males exhibiting a higher incidence than females [5, 6]. Recent years have witnessed an overall rise in melanoma incidence, with varying growth rates observed across different age cohorts [6]. Previously, the primary risk factor for the development of tumors was believed to be excessive exposure to ultraviolet rays [7]. Subsequent research has revealed that the presence of melanocytic nevi, family history, and genetic susceptibility are also significant risk factors [8]. Tumorigenesis is a multifactorial process involving the intricate interplay of various factors, including genes, epigenetics, and the environment [9]. The primary treatment approach for melanoma entails wide local excision, which may be complemented by chemotherapy and targeted therapy. Only a few areas that could not be operated on are treated with radiation therapy due to the radio resistance of melanoma [10].

The immune system assumes a pivotal role in the initiation of tumor formation [11]. During the early stages, melanoma impedes the elimination of cancer cells through two mechanisms of tumor immune evasion. Subsequently, in the advanced stages, cancer cells detach from the primary site via tumor invasion and metastasize to distant regions of the body [12, 13], thereby exacerbating the disease and influencing the prognosis. Tumor infiltrating lymphocytes serve as a crucial role in the immune system’s defense against tumor metastasis [14]. Melanoma exhibits a high frequency of genetic mutations [15, 16], resulting in the generation of neoantigens that trigger the host’s immune response against the tumor. These neoantigens can be utilized for diagnostic, therapeutic, and prognostic purposes [17]. Consequently, personalized care and treatment strategies can be tailored to enhance patients’ physical and psychological well-being and facilitate their recovery.

This study conducted an analysis of immune genes linked to melanoma, resulting in the identification of six genes that were utilized to develop a prognostic model aimed at personalized treatment and prognosis enhancement. The accuracy of this prognostic model was also verified. In addition, the analysis has also revealed that the immune system might regulate the prognosis of melanoma through ‘the regulation of lymphocyte activation’, ‘activation of T cell’, ‘cytotoxicity of NK cell’, and ‘differentiation of Th1 and Th2 cell’ pathways.

## MATERIALS AND METHODS

### Data and patients

1793 genes associated with immunity were extracted from the Immunology Database and Analysis Portal (Immport, http://www.immport.org/home). The RNA-sequencing (RNA-seq) data of melanoma Fragments Per Kilobase Million (FPKM) in the Cancer Genome Atlas (TCGA) was obtained from the Xena database [18] and the amount of 455 melanoma patients with complete survival data was screened based on the relative information of patients.

### Data analysis

According to patients’ gene expression level and survival information, we identified genes that were relevant to prognosis of melanoma from 1793 immune-related genes via Cox univariate analysis, and then we establish a model which could presage the patients’ prognosis of melanoma by combining the Least Absolute Shrinkage and Selection Operator (LASSO) analysis [19, 20] and Cox multivariate analysis (Supplementary Material 1).

### Differential expression analysis

Depending on the prognostic model, risk scores were performed for the patients, and we divided 455 people with melanoma into high risk score (RS) and low RS groups stood on the median of risk scores. The analysis of differential expression was executed on the two groups through the “LIMMA” package [21], and enrichment analysis of differentially expressed genes was executed by the “Clusterprofiler” package [22, 23] (|logFC≥1, P<0.05|).

The miRNA data set of TCGA’s melanoma was downloaded from Xena database, and people with melanoma were split into two groups following the above groups. Differential expression analysis was performed to construct the ceRNA network according to the principle of the ceRNA hypothesis [24] and the miRWalk [25, 26] database (|logFC≥0.7, P<0.05|).

The gene mutation data set of TCGA’s melanoma was downloaded from Xena database, and samples were split into two groups by risk scores. The different of mutation between high RS group and low RS group was analyzed by the “maftools” package [27].

### Immune infiltration and treatment

Estimation of Stromal and Immune cells in malignant tumor tissues using Expression data (ESTIMATE) is a biological information software. In the cancer microenvironment, immune cells and stromal cells are the main normal cells in tumor tissues, and ESTIMATE can use gene expression characteristics to speculate the contents of tumor cells and different infiltrations of normal cells [28].

The tumor microenvironment is closely related to the immunotherapy efficacy of tumor patients [29]. Researchers have classified the tumor microenvironment (TME) into four categories using 29 different functional gene sets: immune-enriched, non-fibrotic (IE); immune-enriched, fibrotic (IE/F); fibrotic (F); and immune-depleted (D) [30].

CIBERSORT (https://cibersort.stanford.edu/) is a method on the ground of linear support vector regression, which can describe the abundance of different cell subsets in complicated tissues from gene expression profiles [31].

Tumor Immune Dysfunction and Exclusion (TIDE) is the tool to estimate the potential of tumor immunologic escape from the gene expression profiles of cancer samples. The score of TIDE computed for each sample can predict response to immune checkpoint blockade as a biomarker.

Patients in the high RS group and the low RS group were evaluated by the ESTIMATE score, and their stromal cell immune cells and tumor purity were obtained in the light of their gene expression. And we explored the relationship between risk score and TME. Then the CIBERSORT analysis was fulfilled on the two groups to compare 22 kinds of immune cells’ infiltration. The analysis of TIDE was used to predict the efficacy of immunotherapy in the two groups. Statistical significance was examined using Students t -tests.

### Validation

The GEO database GSE65904 and GSE19234 were chosen as validation data sets [13, 32]. Then people with melanoma were divided into two groups depended on the model of prognosis, and survival analysis and ROC test on the high RS group and the low RS group were performed to verify the effectiveness of the model.

## RESULTS

### Establishment of prognostic model

**Figure 1:**
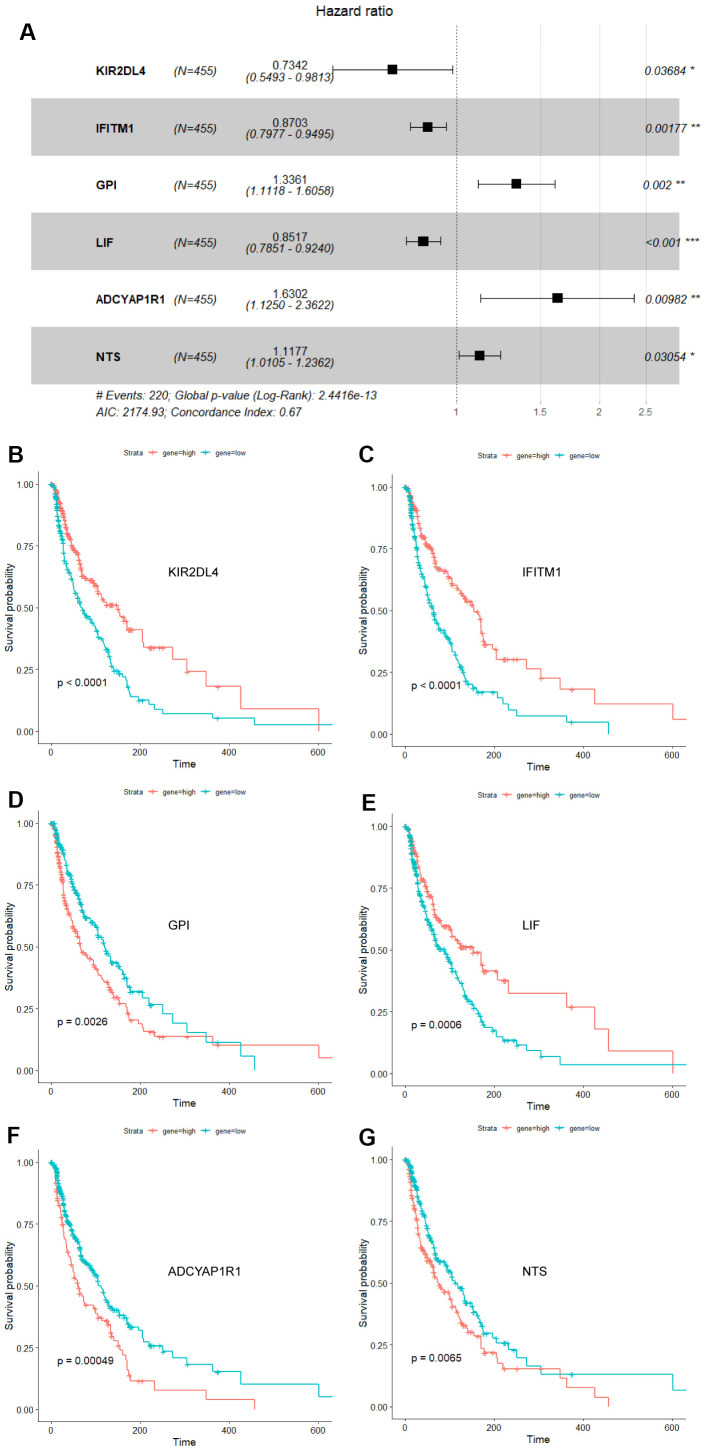
**Forest plot and survival analysis of biomarkers.** (**A**) The Cox proportional hazards model based on ADCYAP1R1, GPI, IFITM1, KIR2DL4, LIF and NTS. (**B**) Survival analysis of KIR2DL4 in melanoma (P<0.0001). (**C**) Survival analysis of IFITM1 in melanoma (P<0.0001). (**D**) Survival analysis of GPI in melanoma (P=0.0026). (**E**) Survival analysis of LIF in melanoma (P=0.0006). (**F**) Survival analysis of ADCYAP1R1 in melanoma (P=0.00049). (**G**) Survival analysis of NTS in melanoma (P=0.0065).

Cox univariate regression analysis was availed to select genes corresponding to the prognosis of melanoma from 1793 immune-related genes, while Lasso regression analysis to further curtail the number of genes. And then, Cox multivariate regression analysis was used for analyzing the outcome of melanoma. Six genes closely associated with the prognosis of melanoma patients, including ADCYAP1R1, GPI, IFITM1, KIR2DL4, LIF and NTS, were elected to fabricate a prognosis model. The calculation equation of the risk score is as shown below: risk score= (-0.30899*KIR2DL4) + (-0.13892*IFITM1) + (0.28977*GPI) + (-0.16050*LIF) + (0.48867* ADCYAP1R1) + (0.11124*NTS). The consistency index (CI) of this model was 0.67 (Figure 1A). The survival curves of these six biomarkers were consistent with the positive and negative coefficients in the formula (Figure 1B–1G).

### Risk score and ROC curve analysis

**Figure 2:**
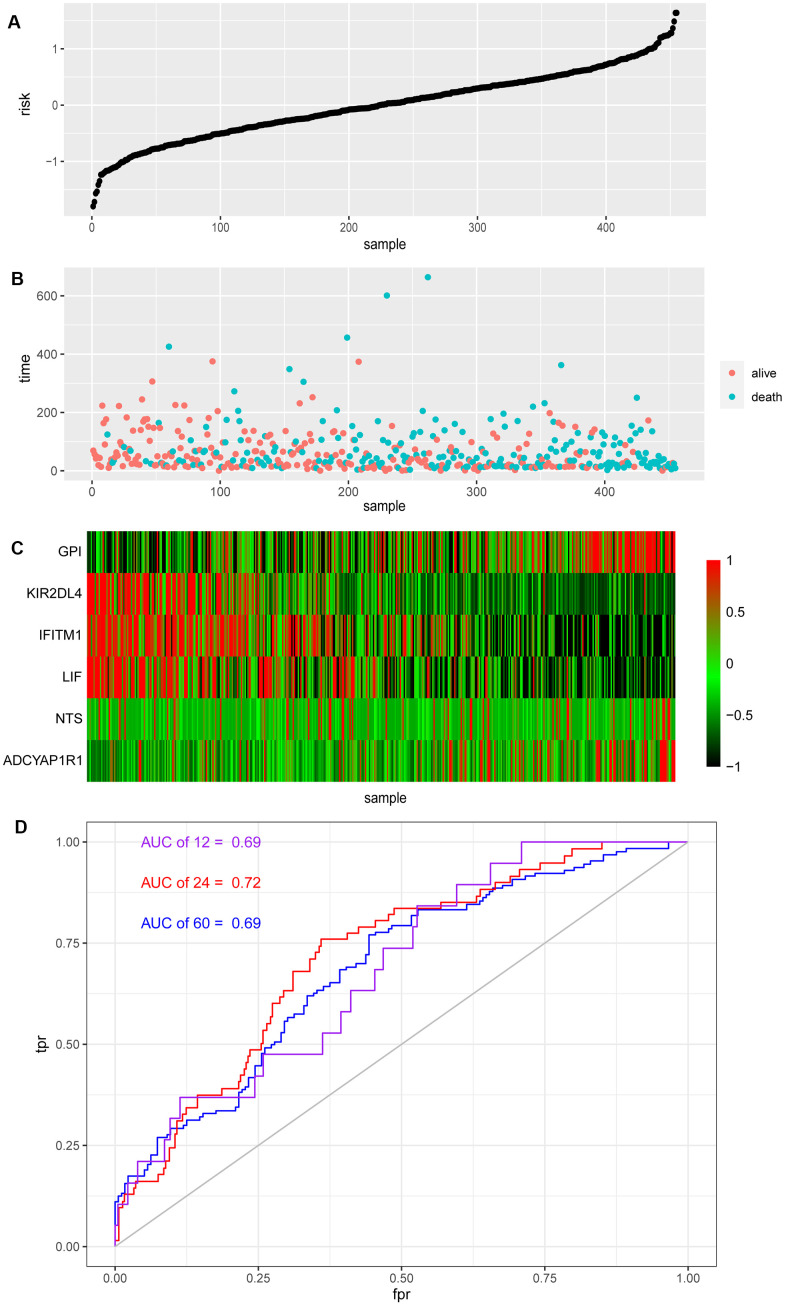
**Risk score and ROC curve of melanoma.** (**A**) Risk score of melanoma patients distributed in ascending order. (**B**) Survival time and status of melanoma patients in order of increasing risk score. The red dots represent the surviving patients and the blue dots represent dead. (**C**) The heatmap shows the expression of these six biomarkers in melanoma in order of increasing risk scores. (**D**) The ROC curve for 1, 2, 5-year survival prediction with AUC value.

Each patient received their risk score based on the above model, while their survival situation was marked, and the expression of each gene in the model from patients was shown by a heat map (Figure 2A–2C). Obviously, ADCYAP1R1, GPI and NTS might lead to poor prognosis for melanoma patients, while IFITM1, KIR2DL4 and LIF are more likely to improve outcomes. At the same time, we also analyzed the effectiveness of the model by the ROC time-dependent curve, within the Area Under the Curve (AUC) of 12, 24 and 60 months scored of 0.69, 0.72 and 0.69, respectively (Figure 2D).

### Enrichment analysis

**Figure 3:**
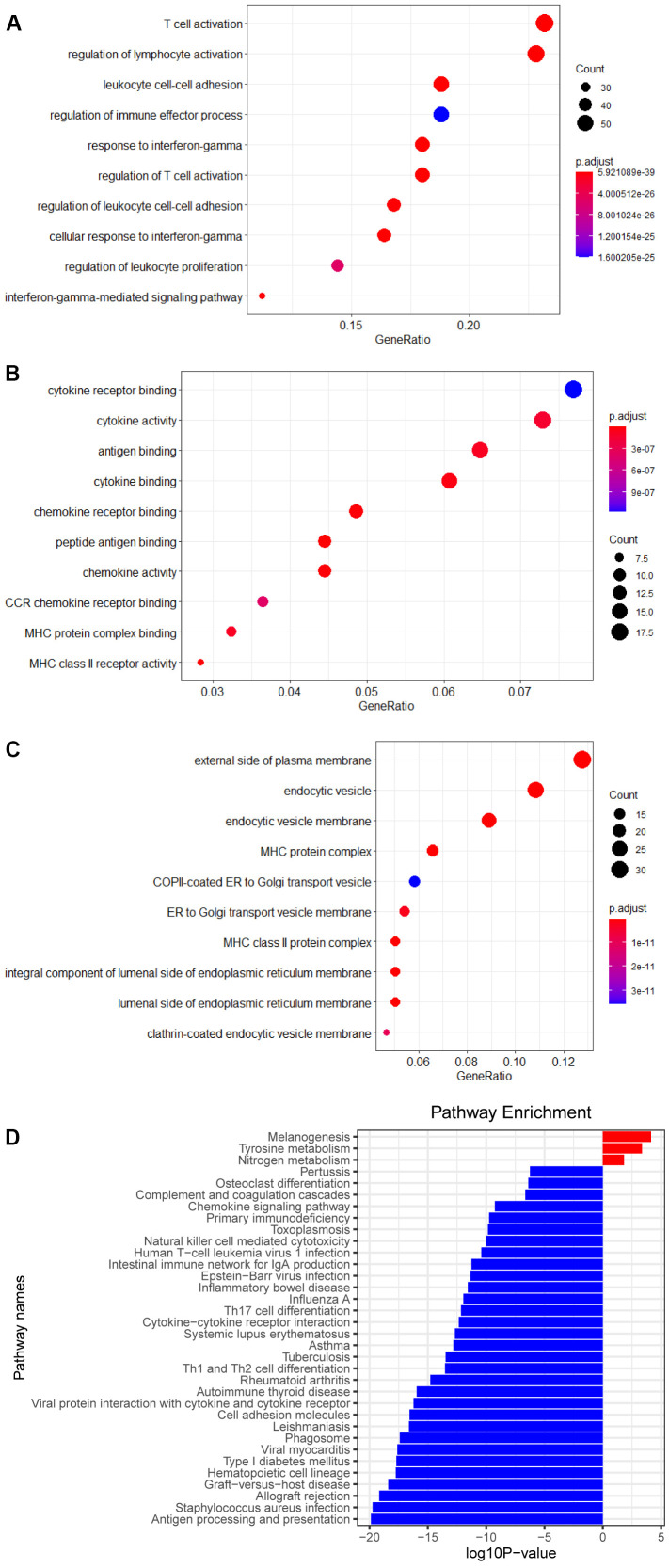
**Enrichment analysis of DEGs.** (**A**) Biological process of DEGs. (**B**) Molecular function of DEGs. (**C**) Cellular component of DEGs. (**D**) KEGG pathways of DEGs.

The risk score which each patient with melanoma received respectively was sorted from smallest to largest, and in consonance with the median, the patients were separated into two groups, a high RS group and a low RS group. 14 genes highly expressed in the high RS group and 249 genes in the low RS group were chalked up through analysis of gene differential expression (| log FC |≥1, *P*<0.05). Enrichment analysis of these genes have shown that the pathways are mainly enriched in ‘the regulation of lymphocyte activation’, ‘activation of T cell’, ‘cytotoxicity of NK cell’, and ‘differentiation of Th1 and Th2 cell’ pathways (Figure 3).

### CeRNA network and somatic mutations

**Figure 4:**
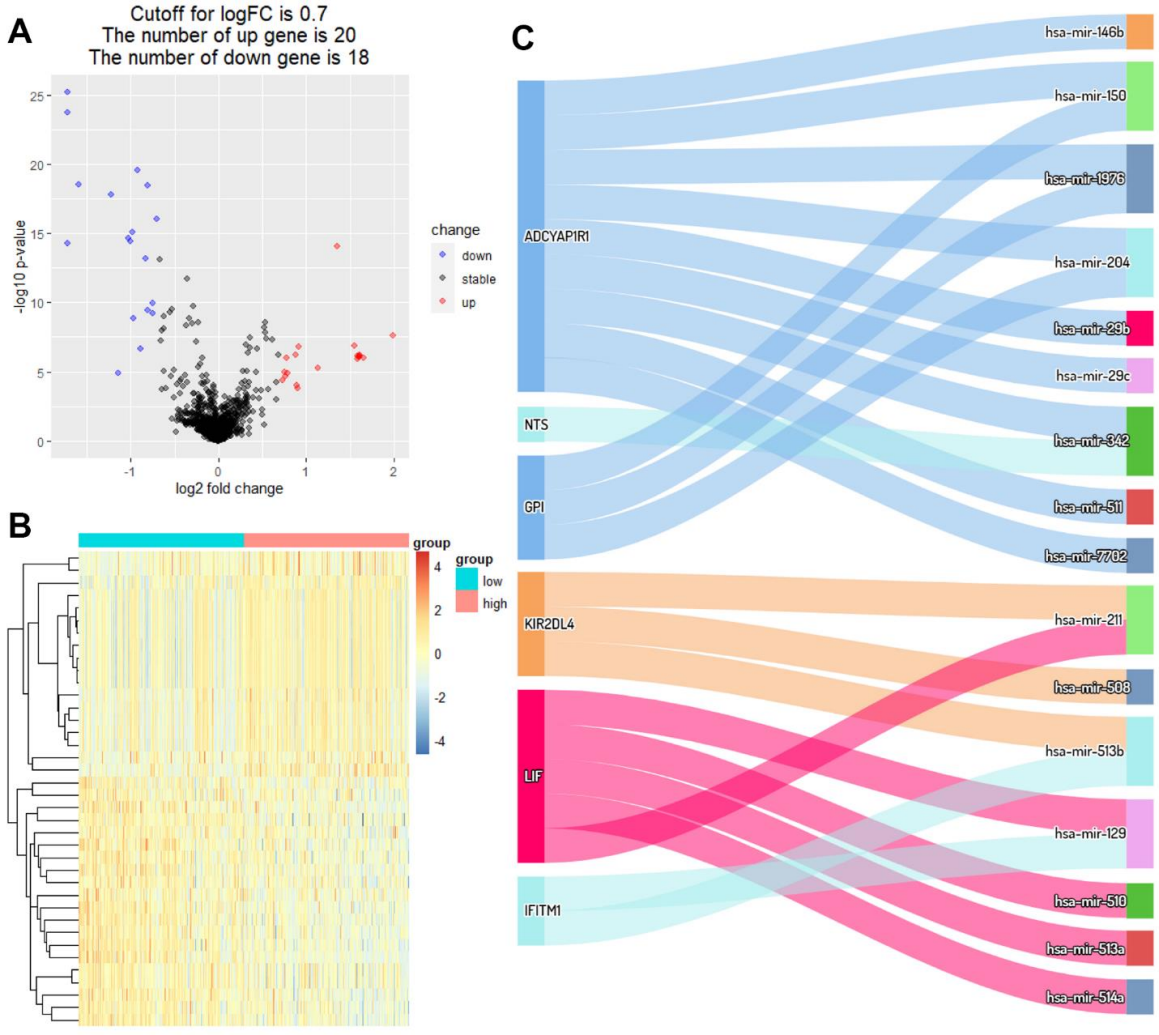
**Identification of DEmiRNAs and ceRNA network.** (**A**) Volcano plot of miRNAs between high RS and low RS groups. (**B**) Heatmap plot of miRNAs between high RS and low RS groups. (**C**) Differential expression of miRNAs-mRNAs network in melanoma.

Hinged on the above risk score grouping, the samples from the miRNA dataset were diverged into the high RS and low RS group for differential expression analysis. 20 miRNAs strongly expressed in the high RS group and 18 miRNAs in the low RS group were screened by analysis of gene differential expression (| log FC |≥0.7, *P*<0.05) (Figure 4A, 4B). According to the theory of ceRNA hypothesis and miRWalk, targeted relationships between miRNAs with differential expression and these 6 biomarkers were found (Figure 4C).

**Figure 5:**
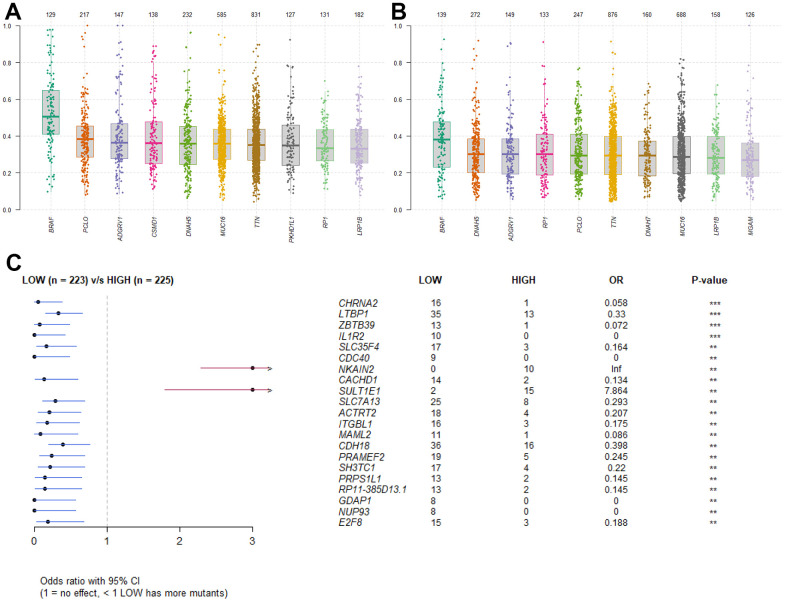
**Somatic mutation analysis.** (**A**) Boxplot of VAF in high RS group. (**B**) Boxplot of VAF in low RS group. (**C**) Forestplot of mutant genes in different groups.

In order to identify the difference of somatic mutations between the two sets, mutation data were envisaged by the “maftools” package in R software [27]. The Variant Allele Frequencies of top mutated genes from the high RS group were stronger than those in the low RS group, suggesting the low-risk patients have better prognosis (Figure 5A, 5B). And the forest plot showed specific mutant genes in two groups (Figure 5C).

### Immune infiltration analysis

**Figure 6:**
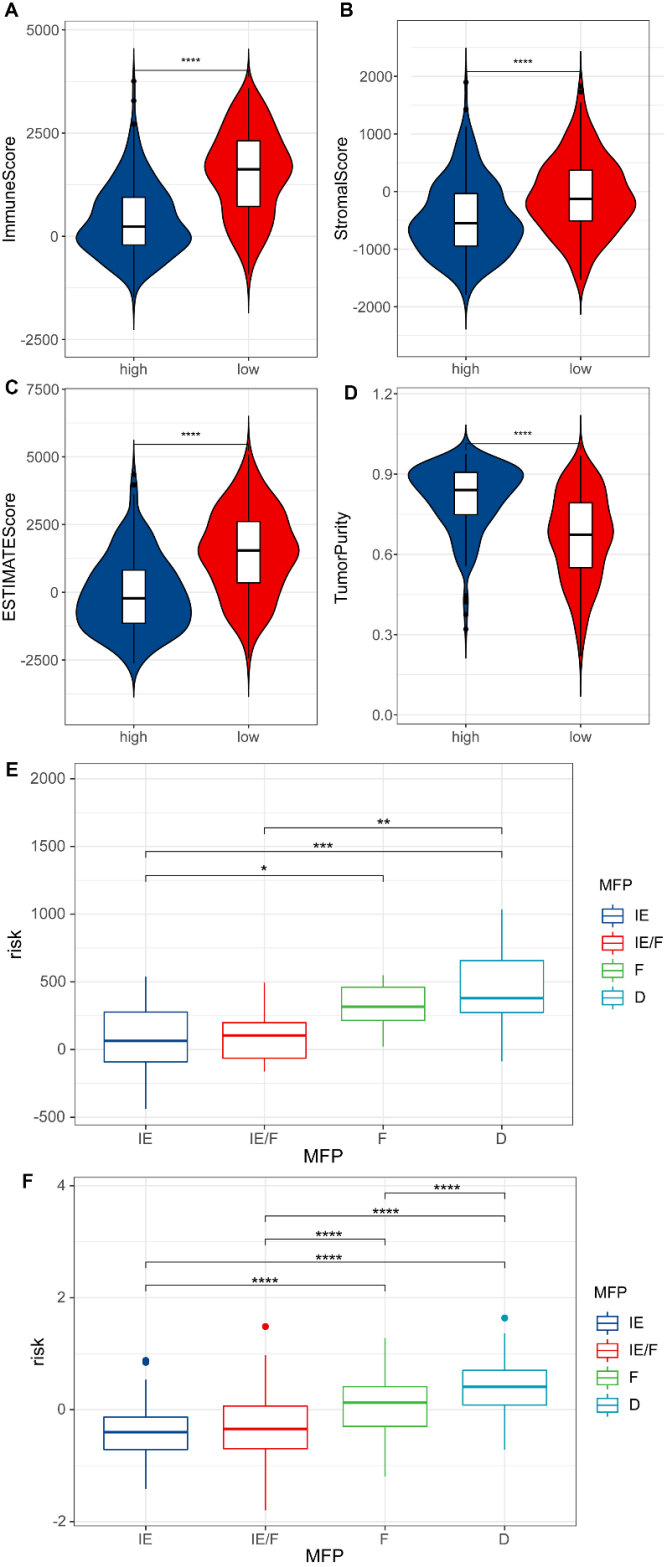
**ESTIMATE analysis.** (**A**) Immune score of high RS samples and low RS samples by ESTIMATE. (**B**) Stromal score of high RS samples and low RS samples by ESTIMATE. (**C**) ESTIMATE score of high RS samples and low RS samples by ESTIMATE. (**D**) Tumor purity of high RS samples and low RS samples by ESTIMATE. (**E**) Risk scores for four TME subtypes in samples of GSE22153. (**F**) Risk scores for four TME subtypes in melanoma samples of TCGA.

In the above two groups which were separated by ESTIMATE analysis, it was found that the low RS group might have a better Immune score, Stromal score and ESTIMATE score than the high RS group, while the tumor purity was the opposite (P <0.0001) (Figure 6A–6D), which demonstrated the reliability of our model and the strong correlation between the model and the patients’ immune infiltrate.

**Figure 7:**
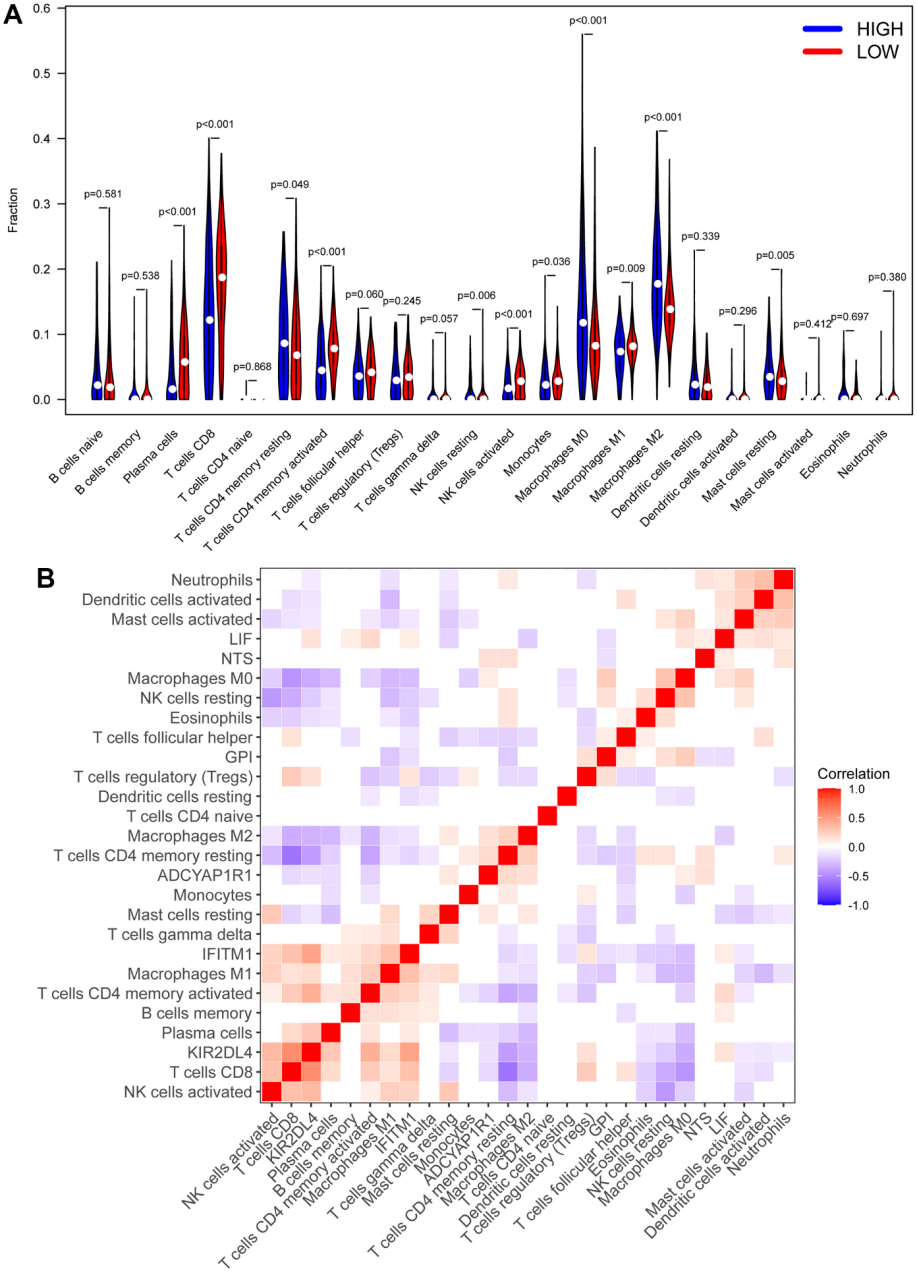
**CIBERSORT analysis.** (**A**) The heatmap of immune cell infiltration in high RS samples and low RS samples. Red represents low RS samples, and blue represents high-risk samples. (**B**) Correlation analysis between different immune cells and biomarkers.

The TME can be divided into four subtypes: immune-enriched, non-fibrotic (IE); immune-enriched, fibrotic (IE/F); fibrotic (F); and immune-depleted (D). We calculated risk scores for melanoma patients in GSE22153 and TCGA using the prognostic model. It was found that IE and IE/F groups have lower risk scores compared to the F and D groups (Figure 6E, 6F).

cibersort analysis was performed for immune infiltration of patients from the high- and low RS group. We found that patients in the low RS group had high expression in plasma cells, T cell CD8^+^, T cell CD4^+^ memory activated, and NK cells activated, while Macrophages M0 and Macrophages M2 were strongly expressed in the high RS group (Figure 7A). Moreover, we also analyzed the correlation between the 6 biomarkers and immune cells, and found that KIR2DL4 was related to T cell CD8^+^, T cell CD4^+^ memory activated, and NK cells activated (Figure 7B).

**Figure 8:**
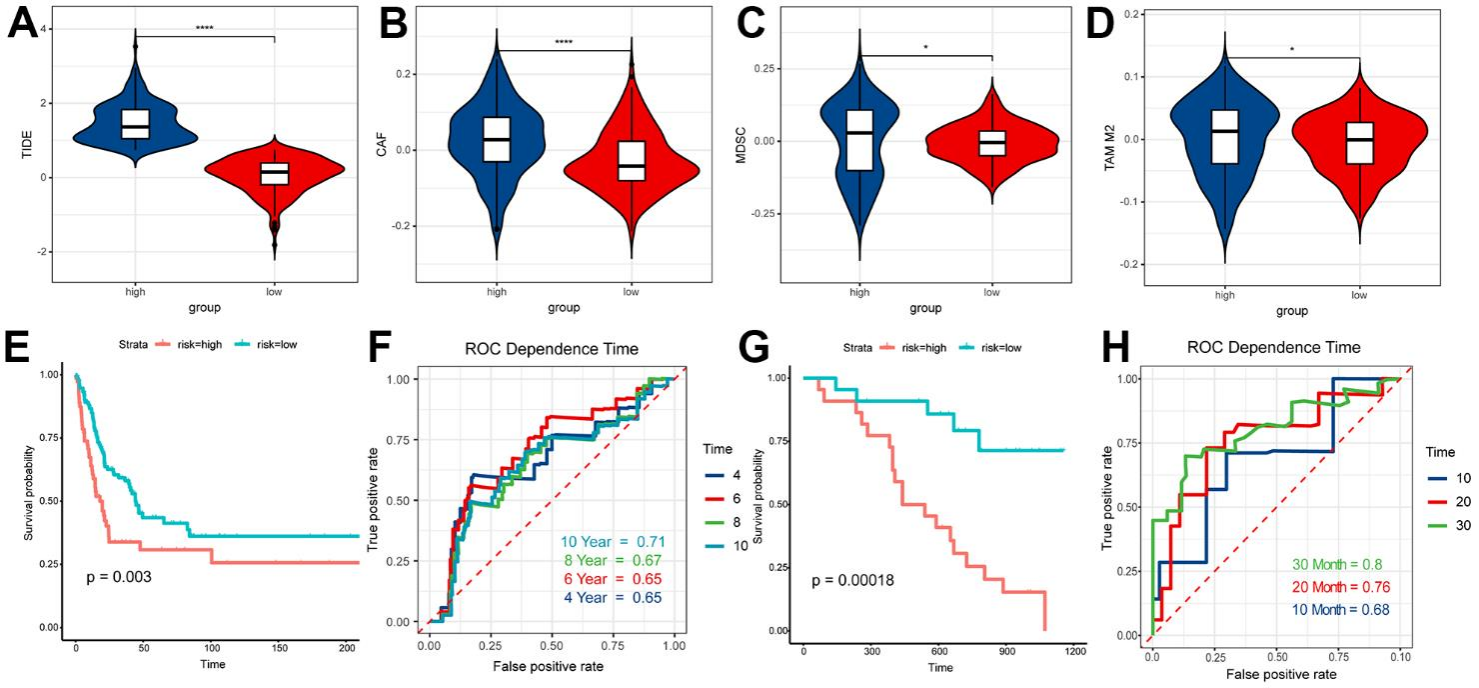
**TIDE analysis and validation.** (**A**) TIDE score of high RS samples and low RS samples. (**B**) CAF score of high RS samples and low RS samples. (**C**) MDSC score of high RS samples and low RS samples. (**D**) TAM M2 score of high RS samples and low RS samples. (**E**) Survival analysis of risk score in GSE65904. (**F**) The ROC curve for 4, 6, 8, 10-year survival prediction with AUC value. (**G**) Survival analysis of risk score in GSE19234. (**H**) The ROC curve for 10, 20, 30-month survival prediction with AUC value.

According to the results of ESTIMATE, TME and CIBERSORT, the difference of prognosis between the above two groups might be closely associated with T cells. Thus, we used the TIDE to evaluate the therapeutic efficaciousness of immune checkpoint suppression in the two groups. The TIDE score of patients in the high RS group had a better score than those in low one (Figure 8A). The scores of the M2 subtype of tumor-associated macrophages (TAMs), myeloid-derived suppressor cells (MDSCs) and cancer-associated fibroblasts (CAFs) in the high RS group were higher than the low one (Figure 8B–8D). All results showed that the patients in low RS group had greater prognosis under immune checkpoint inhibition therapy.

### Experimental validation

In this study, samples of GSE65904 and GSE19234 were graded according to the prognostic model, and the scoring results were sorted from lowest to highest, and based on the median, the people participated in our research were split into two groups, the high RS group and the low RS group. The survival analysis of the two groups based on survival information suggested that people in the low RS group have significantly better outcomes than people in another group (*P <* 0.01) (Figure 8E, 8G). The ROC time-dependent curve was applied to verify the prognostic model and all AUCs were greater than or equal to 0.65 (Figure 8F, 8H). These results all validated the effectiveness of our model.

## DISCUSSION

Melanoma, as a highly malignant tumor derived from melanocytes, accounts for about 3% of all tumors, mainly occurring in the skin, and a few in the mucosa and viscera [33]. Among the most common malignant tumors, the incidence of cutaneous malignant melanoma is the third (6.8%~20%) [34], and during these years, the morbidity and mortality have been still increasing. Compared with other solid tumors, malignant melanoma makes the clinical prognosis worse, with a median survival time of about 6 months and a 1-year overall survival rate (OS) of 25% [35]. Immune checkpoint inhibitor (ICI) has been presented to significantly boost overall survival rate in persons with advanced-stage melanoma. Ipilimumab (the anti-CTLA-4 antibodies) and nivolumab (the anti-PD-1 antibodies) serve as the routine treatment for advanced melanoma nowadays [36]. In this study, six genes (ADCYAP1R1, GPI, IFITM1, KIR2DL4, LIF, and NTS) that exhibited strong correlation with the prognosis of melanoma patients were identified. These genes were utilized to develop a prognostic model that demonstrates reliable predictive value and accuracy. Additionally, the model has the capability to predict the prognosis of ICI therapy. Moreover, notable differences were observed in the infiltration of immune cells and activation of multiple signaling pathways between the two groups.

In this study, six genes (ADCYAP1R1, GPI, IFITM1, KIR2DL4, LIF, and NTS) were identified as being closely associated with prognosis among a pool of 1793 melanoma-related genes. This selection was achieved through the implementation of Cox univariate regression analysis, Lasso analysis, and Cox multivariate regression analysis, ultimately leading to the establishment of a prognostic model. Subsequently, individuals diagnosed with melanoma were categorized into either a high-risk score (RS) group or a low RS group based on this model, and its efficacy was subsequently validated. Our findings indicate that ADCYAP1R1, GPI, and NTS may contribute to a poor prognosis in melanoma patients, while IFITM1, KIR2DL4, and LIF are more likely to be associated with a better prognosis in individuals with melanoma. These genes have been manifested to be involved in the tumorigenesis in precedent studies. ADCYAP1R1 (ADCYAP Receptor Type I), a Protein Coding gene, involves in related pathways including synthesis and secretion of Aldosterone as well as Signaling mediated by G-protein coupled receptor (GPCR). GO annotations associated with this gene include activities of G protein-coupled receptor and trans membrane signaling receptor [37]. The key role of LGR4, a member of the GPCR family, has been demonstrated in tumor immunoregulation, and tumor immunotherapy strategies targeting LGR4 have been proposed and validated. Inhibition of Rspo-Lgr4 switches the polarization of macrophage in order to facilitate checkpoint blockade therapy [38]. GPI can encode the protein that is a member of the glucose phosphate isomerase protein family which has been determined as a moonlighting protein, because it is capable to execute distinct functions mechanistically [39]. The encoded protein, plays a role as a neurotrophic factor extracellularly, which can promote survival of skeletal motor and sensory neurons, and also as a lymphokine to induce secretion of immunoglobulin [40]. According to the auxiliary function of tumor-secreted cytokine and angiogenic factor, it is also as an autocrine motility factor [41]. Neuromedin N and neurotensin have a common precursor, which is encoded by NTS [42]. Neurotensin is a secreted tripeptide extensively distributed in the central nervous system and it may be involved in maintenance of intestinal structure and function, and regulation of fat metabolism [43]. Diseases associated with IFITM1 include Influenza and Dengue Virus [44]. Its related pathways are Interferon gamma signaling [45] and Innate Immune System [46]. GO annotations connected with this gene include obsolete signal transducer activity, downstream of receptor [47]. Killer cell immunoglobulin-like receptors (KIRs) are expressed by NK cells and subsets of T cells as transmembrane glycoproteins [48]. KIR proteins are deemed to play a part in regulation of the immune response because the ligands belonging to subsets of HLA class molecules [49]. Leukemia and Myeloid Leukemia are associated with LIF (Interleukin 6 Family Cytokine), which can code related protein [50]. Among its related pathways is Interleukin-6 family signaling [51]. Therefore, the above studies suggested the rationality and feasibility of the screened 6 genes in the prediction of melanoma incidence and prognosis.

The analysis of differential expression on the genes of patients in the two groups was completed, resulting in the identification of 14 genes highly expressed in the high RS group and 249 genes in the other group. The results of pathway enrichment analysis demonstrated that these genes were mainly associated with several pathways including lymphocyte activation regulation, activation of T cell, cytotoxicity of NK cell, and differentiation of Th1 and Th2 cell. In contrast to T cells, which exhibit specificity towards a singular aberrant molecule on cancerous cells and initiate a targeted assault, natural killer (NK) cells have demonstrated their versatility in the initial defense against cancer. NK cells not only serve as crucial effector cells in the innate immune response, but also function as regulators of diverse immune cell populations. Joy Hsu et al. [52] found that the level of PD-L1-positive NK cells was specifically connected with the outcome of patients with cancer after analyzing the NK cells in human and mice (tumor cells were PD-L1-negative). Under the action of immune checkpoint inhibitors, PD-L1-positive NK cells can not only eliminate tumor cells immediately, but also secrete cytokines to control tumor growth. Combined with NK cell activating factor on the basis of PD-L1 antibody can also significantly improve the therapeutic effect. Therefore, NK cells still have more potential to be explored in tumor therapy. Previous studies have shown that in tumor microenvironment (TME), immune infiltration is vital in tumor genesis and progression, and affects the clinical prognosis of tumor patients [53, 54]. In this study, we found that the melanoma prognostic model constructed using these 6 genes was highly correlated with the patient’s immune infiltration. To explore the correlation with circumstance on immune cell infiltration deeply, we continued to use CIBERSORT analysis to compare patients in the two groups, and found that different groups expressed different immune cell subtypes. People in the low RS group showed high expression in plasma cells, T cell CD8^+^, T cell CD4^+^ memory activated, and NK cells activated, while Macrophages M0 and Macrophages M2 were highly expressed in another group. Ali et al. [55, 56] have shown that the imbalance of the proportion of immune cell components has strongly correlation with poor outcome and low survival rate of patients with cancer. The past studies have covered that T cell CD8^+^ and NK cells perform a vital role in tumor immunity [57]. Furthermore, an examination was conducted to investigate the association between the aforementioned 6 biomarkers and immune cells. The results revealed a significant correlation between KIR2DL4 and T cell CD8^+^, T cell CD4^+^ memory activated, as well as NK cells activated. It is suggested that the abundant expression of T cell CD8^+^ and NK cells might reduce the risk elements related to melanoma and ameliorate the prognosis of sick people.

The ICI therapy is the main treatment in the current therapeutic method of melanoma which can significantly upgrade the overall survival rate of people with advanced melanoma [36]. CAFs, MDSCs and TAMs can restrict infiltration of T lymphocyte in tumors [58]. In the study, we found that the scores of CAFs, MDSCs and TAMs in the high RS group were better than those in another group, which means the people with high risk have higher T cell exclusion and lower infiltration of cytotoxic T lymphocytes. Although the ICI enhance the survival rate of skin melanoma patients, only some patients could be benefited from the ICI treatment [59]. Meanwhile, patients are also faced with heavy economic and psychological burden in the treatment process. The TIDE score could presage the response to ICI therapy [60]. A positive correlation is observed between lower TIDE scores and improved prognosis. Our study reveals that the low-risk group exhibits lower TIDE scores, indicating potential benefits from ICI therapy. Consequently, the model may serve as a valuable tool for determining the suitability of ICI treatment.

Our research inevitably presents opportunities for further improvement. Firstly, the study entails a bioinformatics analysis utilizing publicly available databases, thereby limiting the authenticity of the molecular mechanism analysis results due to the absence of *in vivo* or *in vitro* experiments. Secondly, the sample data obtained from the public database is subject to restrictions, potentially introducing random errors. Moreover, our study only provides some new potential research targets for the prognosis and treatment of melanoma. But the deeper molecular mechanism still needs to be further explored.

## CONCLUSIONS

In our study, an effective prognostic model for melanoma was established. All bioinformatics results demonstrated the potential of the six key genes as prognostic markers of melanoma patients and the strong correlation of the model with immune infiltration. This study has provided more information for the pathogenesis and clinical treatment of melanoma.

## Supplementary Material

Supplementary Material 1

## References

[1] Islami F, Ward EM, Sung H, Cronin KA, Tangka FK, Sherman RL, Zhao J, Anderson RN, Henley SJ, Yabroff KR, Jemal A, Benard VB. Annual Report to the Nation on the Status of Cancer, Part 1: National Cancer Statistics. J Natl Cancer Inst. 2021; 113:1648–69. 10.1093/jnci/djab131 34240195 PMC8634503

[2] Weiss SA, Wolchok JD, Sznol M. Immunotherapy of Melanoma: Facts and Hopes. Clin Cancer Res. 2019; 25:5191–201. 10.1158/1078-0432.CCR-18-1550 30923036 PMC6726509

[3] Feigelson HS, Powers JD, Kumar M, Carroll NM, Pathy A, Ritzwoller DP. Melanoma incidence, recurrence, and mortality in an integrated healthcare system: A retrospective cohort study. Cancer Med. 2019; 8:4508–16. 10.1002/cam4.2252 31215776 PMC6675720

[4] Siegel RL, Miller KD, Jemal A. Cancer statistics, 2019. CA Cancer J Clin. 2019; 69:7–34. 10.3322/caac.21551 30620402

[5] Rastrelli M, Tropea S, Rossi CR, Alaibac M. Melanoma: epidemiology, risk factors, pathogenesis, diagnosis and classification. In Vivo. 2014; 28:1005–11. 25398793

[6] Guy GP Jr, Thomas CC, Thompson T, Watson M, Massetti GM, Richardson LC, and Centers for Disease Control and Prevention (CDC). Vital signs: melanoma incidence and mortality trends and projections - United States, 1982-2030. MMWR Morb Mortal Wkly Rep. 2015; 64:591–6. 26042651 PMC4584771

[7] Hawkes JE, Truong A, Meyer LJ. Genetic predisposition to melanoma. Semin Oncol. 2016; 43:591–7. 10.1053/j.seminoncol.2016.08.003 27899192

[8] Leonardi GC, Falzone L, Salemi R, Zanghì A, Spandidos DA, Mccubrey JA, Candido S, Libra M. Cutaneous melanoma: From pathogenesis to therapy (Review). Int J Oncol. 2018; 52:1071–80. 10.3892/ijo.2018.4287 29532857 PMC5843392

[9] Davey MG, Miller N, McInerney NM. A Review of Epidemiology and Cancer Biology of Malignant Melanoma. Cureus. 2021; 13:e15087. 10.7759/cureus.15087 34155457 PMC8210625

[10] Trappetti V, Fazzari JM, Fernandez-Palomo C, Scheidegger M, Volarevic V, Martin OA, Djonov VG. Microbeam Radiotherapy-A Novel Therapeutic Approach to Overcome Radioresistance and Enhance Anti-Tumour Response in Melanoma. Int J Mol Sci. 2021; 22:7755. 10.3390/ijms22147755 34299373 PMC8303317

[11] Gajewski TF, Schreiber H, Fu YX. Innate and adaptive immune cells in the tumor microenvironment. Nat Immunol. 2013; 14:1014–22. 10.1038/ni.2703 24048123 PMC4118725

[12] Lee N, Zakka LR, Mihm MC Jr, Schatton T. Tumour-infiltrating lymphocytes in melanoma prognosis and cancer immunotherapy. Pathology. 2016; 48:177–87. 10.1016/j.pathol.2015.12.006 27020390

[13] Cabrita R, Lauss M, Sanna A, Donia M, Skaarup Larsen M, Mitra S, Johansson I, Phung B, Harbst K, Vallon-Christersson J, van Schoiack A, Lövgren K, Warren S, et al. Tertiary lymphoid structures improve immunotherapy and survival in melanoma. Nature. 2020; 577:561–5. 10.1038/s41586-019-1914-8 31942071

[14] Borgers JSW, Haanen JB. Cellular Therapy and Cytokine Treatments for Melanoma. Hematol Oncol Clin North Am. 2021; 35:129–44. 10.1016/j.hoc.2020.08.014 33759770

[15] Nassar KW, Tan AC. The mutational landscape of mucosal melanoma. Semin Cancer Biol. 2020; 61:139–48. 10.1016/j.semcancer.2019.09.013 31655118 PMC7078020

[16] Ribero S, Glass D, Bataille V. Genetic epidemiology of melanoma. Eur J Dermatol. 2016; 26:335–9. 10.1684/ejd.2016.2787 27436815

[17] Jiang J, Ding Y, Wu M, Chen Y, Lyu X, Lu J, Wang H, Teng L. Integrated genomic analysis identifies a genetic mutation model predicting response to immune checkpoint inhibitors in melanoma. Cancer Med. 2020; 9:8498–518. 10.1002/cam4.3481 32969604 PMC7666739

[18] Goldman MJ, Craft B, Hastie M, Repečka K, McDade F, Kamath A, Banerjee A, Luo Y, Rogers D, Brooks AN, Zhu J, Haussler D. Visualizing and interpreting cancer genomics data via the Xena platform. Nat Biotechnol. 2020; 38:675–8. 10.1038/s41587-020-0546-8 32444850 PMC7386072

[19] Vasquez MM, Hu C, Roe DJ, Halonen M, Guerra S. Measurement error correction in the least absolute shrinkage and selection operator model when validation data are available. Stat Methods Med Res. 2019; 28:670–80. 10.1177/0962280217734241 29166842 PMC7449511

[20] Friedman J, Hastie T, Tibshirani R. Regularization Paths for Generalized Linear Models via Coordinate Descent. J Stat Softw. 2010; 33:1–22. 10.18637/jss.v033.i01 20808728 PMC2929880

[21] Ritchie ME, Phipson B, Wu D, Hu Y, Law CW, Shi W, Smyth GK. limma powers differential expression analyses for RNA-sequencing and microarray studies. Nucleic Acids Res. 2015; 43:e47. 10.1093/nar/gkv007 25605792 PMC4402510

[22] Yu G, Wang LG, Han Y, He QY. clusterProfiler: an R package for comparing biological themes among gene clusters. OMICS. 2012; 16:284–7. 10.1089/omi.2011.0118 22455463 PMC3339379

[23] Fang K, Xu JX, Chen XX, Gao XR, Huang LL, Du AQ, Jiang C, Ge JF. Differential serum exosome microRNA profile in a stress-induced depression rat model. J Affect Disord. 2020; 274:144–58. 10.1016/j.jad.2020.05.017 32469797

[24] Tay Y, Rinn J, Pandolfi PP. The multilayered complexity of ceRNA crosstalk and competition. Nature. 2014; 505:344–52. 10.1038/nature12986 24429633 PMC4113481

[25] Miao L, Yin RX, Zhang QH, Liao PJ, Wang Y, Nie RJ, Li H. A novel circRNA-miRNA-mRNA network identifies circ-YOD1 as a biomarker for coronary artery disease. Sci Rep. 2019; 9:18314. 10.1038/s41598-019-54603-2 31797949 PMC6892882

[26] Dweep H, Sticht C, Pandey P, Gretz N. miRWalk--database: prediction of possible miRNA binding sites by “walking” the genes of three genomes. J Biomed Inform. 2011; 44:839–47. 10.1016/j.jbi.2011.05.002 21605702

[27] Mayakonda A, Lin DC, Assenov Y, Plass C, Koeffler HP. Maftools: efficient and comprehensive analysis of somatic variants in cancer. Genome Res. 2018; 28:1747–56. 10.1101/gr.239244.118 30341162 PMC6211645

[28] Yoshihara K, Shahmoradgoli M, Martínez E, Vegesna R, Kim H, Torres-Garcia W, Treviño V, Shen H, Laird PW, Levine DA, Carter SL, Getz G, Stemke-Hale K, et al. Inferring tumour purity and stromal and immune cell admixture from expression data. Nat Commun. 2013; 4:2612. 10.1038/ncomms3612 24113773 PMC3826632

[29] Wang Z, Liu Y, Mo Y, Zhang H, Dai Z, Zhang X, Ye W, Cao H, Liu Z, Cheng Q. The CXCL Family Contributes to Immunosuppressive Microenvironment in Gliomas and Assists in Gliomas Chemotherapy. Front Immunol. 2021; 12:731751. 10.3389/fimmu.2021.731751 34603309 PMC8482424

[30] Bagaev A, Kotlov N, Nomie K, Svekolkin V, Gafurov A, Isaeva O, Osokin N, Kozlov I, Frenkel F, Gancharova O, Almog N, Tsiper M, Ataullakhanov R, Fowler N. Conserved pan-cancer microenvironment subtypes predict response to immunotherapy. Cancer Cell. 2021; 39:845–65.e7. 10.1016/j.ccell.2021.04.014 34019806

[31] Oh SC, Sohn BH, Cheong JH, Kim SB, Lee JE, Park KC, Lee SH, Park JL, Park YY, Lee HS, Jang HJ, Park ES, Kim SC, et al. Clinical and genomic landscape of gastric cancer with a mesenchymal phenotype. Nat Commun. 2018; 9:1777. 10.1038/s41467-018-04179-8 29725014 PMC5934392

[32] Bogunovic D, O’Neill DW, Belitskaya-Levy I, Vacic V, Yu YL, Adams S, Darvishian F, Berman R, Shapiro R, Pavlick AC, Lonardi S, Zavadil J, Osman I, Bhardwaj N. Immune profile and mitotic index of metastatic melanoma lesions enhance clinical staging in predicting patient survival. Proc Natl Acad Sci USA. 2009; 106:20429–34. 10.1073/pnas.0905139106 19915147 PMC2787158

[33] Dinnes J, Deeks JJ, Chuchu N, Ferrante di Ruffano L, Matin RN, Thomson DR, Wong KY, Aldridge RB, Abbott R, Fawzy M, Bayliss SE, Grainge MJ, Takwoingi Y, et al, and Cochrane Skin Cancer Diagnostic Test Accuracy Group. Dermoscopy, with and without visual inspection, for diagnosing melanoma in adults. Cochrane Database Syst Rev. 2018; 12:CD011902. 10.1002/14651858.CD011902.pub2 30521682 PMC6517096

[34] Driscoll MS, Martires K, Bieber AK, Pomeranz MK, Grant-Kels JM, Stein JA. Pregnancy and melanoma. J Am Acad Dermatol. 2016; 75:669–78. 10.1016/j.jaad.2016.01.061 27646737

[35] Rosenberg SA, Lotze MT, Yang JC, Topalian SL, Chang AE, Schwartzentruber DJ, Aebersold P, Leitman S, Linehan WM, Seipp CA. Prospective randomized trial of high-dose interleukin-2 alone or in conjunction with lymphokine-activated killer cells for the treatment of patients with advanced cancer. J Natl Cancer Inst. 1993; 85:622–32. 10.1093/jnci/85.8.622 8468720

[36] Marconcini R, Spagnolo F, Stucci LS, Ribero S, Marra E, Rosa F, Picasso V, Di Guardo L, Cimminiello C, Cavalieri S, Orgiano L, Tanda E, Spano L, et al, and Italian Melanoma Intergroup (IMI). Current status and perspectives in immunotherapy for metastatic melanoma. Oncotarget. 2018; 9:12452–70. 10.18632/oncotarget.23746 29552325 PMC5844761

[37] Kobayashi K, Shihoya W, Nishizawa T, Kadji FM, Aoki J, Inoue A, Nureki O. Cryo-EM structure of the human PAC1 receptor coupled to an engineered heterotrimeric G protein. Nat Struct Mol Biol. 2020; 27:274–80. 10.1038/s41594-020-0386-8 32157248

[38] Tan B, Shi X, Zhang J, Qin J, Zhang N, Ren H, Qian M, Siwko S, Carmon K, Liu Q, Han H, Du B, Liu M. Inhibition of Rspo-Lgr4 Facilitates Checkpoint Blockade Therapy by Switching Macrophage Polarization. Cancer Res. 2018; 78:4929–42. 10.1158/0008-5472.CAN-18-0152 29967265

[39] Kainulainen V, Loimaranta V, Pekkala A, Edelman S, Antikainen J, Kylväjä R, Laaksonen M, Laakkonen L, Finne J, Korhonen TK. Glutamine synthetase and glucose-6-phosphate isomerase are adhesive moonlighting proteins of Lactobacillus crispatus released by epithelial cathelicidin LL-37. J Bacteriol. 2012; 194:2509–19. 10.1128/JB.06704-11 22389474 PMC3347177

[40] Mizrachi Y. Neurotrophic activity of monomeric glucophosphoisomerase was blocked by human immunodeficiency virus (HIV-1) and peptides from HIV-1 envelope glycoprotein. J Neurosci Res. 1989; 23:217–24. 10.1002/jnr.490230212 2547084

[41] Nakajima K, Raz A. Amplification of autocrine motility factor and its receptor in multiple myeloma and other musculoskeletal tumors. J Bone Oncol. 2020; 23:100308. 10.1016/j.jbo.2020.100308 32714781 PMC7378681

[42] Tabarean IV. Neurotensin induces hypothermia by activating both neuronal neurotensin receptor 1 and astrocytic neurotensin receptor 2 in the median preoptic nucleus. Neuropharmacology. 2020; 171:108069. 10.1016/j.neuropharm.2020.108069 32275927 PMC7274271

[43] Steele FF 3rd, Whitehouse SC, Aday JS, Prus AJ. Neurotensin NTS1 and NTS2 receptor agonists produce anxiolytic-like effects in the 22-kHz ultrasonic vocalization model in rats. Brain Res. 2017; 1658:31–5. 10.1016/j.brainres.2017.01.012 28089664

[44] Brass AL, Huang IC, Benita Y, John SP, Krishnan MN, Feeley EM, Ryan BJ, Weyer JL, van der Weyden L, Fikrig E, Adams DJ, Xavier RJ, Farzan M, Elledge SJ. The IFITM proteins mediate cellular resistance to influenza A H1N1 virus, West Nile virus, and dengue virus. Cell. 2009; 139:1243–54. 10.1016/j.cell.2009.12.017 20064371 PMC2824905

[45] Yang G, Xu Y, Chen X, Hu G. IFITM1 plays an essential role in the antiproliferative action of interferon-gamma. Oncogene. 2007; 26:594–603. 10.1038/sj.onc.1209807 16847454

[46] Prasad K, Khatoon F, Rashid S, Ali N, AlAsmari AF, Ahmed MZ, Alqahtani AS, Alqahtani MS, Kumar V. Targeting hub genes and pathways of innate immune response in COVID-19: A network biology perspective. Int J Biol Macromol. 2020; 163:1–8. 10.1016/j.ijbiomac.2020.06.228 32599245 PMC7319641

[47] Vishnubalaji R, Shaath H, Elkord E, Alajez NM. Long non-coding RNA (lncRNA) transcriptional landscape in breast cancer identifies LINC01614 as non-favorable prognostic biomarker regulated by TGFβ and focal adhesion kinase (FAK) signaling. Cell Death Discov. 2019; 5:109. 10.1038/s41420-019-0190-6 31263577 PMC6591245

[48] Lunemann S, Schöbel A, Kah J, Fittje P, Hölzemer A, Langeneckert AE, Hess LU, Poch T, Martrus G, Garcia-Beltran WF, Körner C, Ziegler AE, Richert L, et al. Interactions Between KIR3DS1 and HLA-F Activate Natural Killer Cells to Control HCV Replication in Cell Culture. Gastroenterology. 2018; 155:1366–71.e3. 10.1053/j.gastro.2018.07.019 30031767

[49] Pende D, Falco M, Vitale M, Cantoni C, Vitale C, Munari E, Bertaina A, Moretta F, Del Zotto G, Pietra G, Mingari MC, Locatelli F, Moretta L. Killer Ig-Like Receptors (KIRs): Their Role in NK Cell Modulation and Developments Leading to Their Clinical Exploitation. Front Immunol. 2019; 10:1179. 10.3389/fimmu.2019.01179 31231370 PMC6558367

[50] Zhang C, Liu J, Wang J, Hu W, Feng Z. The emerging role of leukemia inhibitory factor in cancer and therapy. Pharmacol Ther. 2021; 221:107754. 10.1016/j.pharmthera.2020.107754 33259884 PMC8084904

[51] Jones SA, Jenkins BJ. Recent insights into targeting the IL-6 cytokine family in inflammatory diseases and cancer. Nat Rev Immunol. 2018; 18:773–89. 10.1038/s41577-018-0066-7 30254251

[52] Dong W, Wu X, Ma S, Wang Y, Nalin AP, Zhu Z, Zhang J, Benson DM, He K, Caligiuri MA, Yu J. The Mechanism of Anti-PD-L1 Antibody Efficacy against PD-L1-Negative Tumors Identifies NK Cells Expressing PD-L1 as a Cytolytic Effector. Cancer Discov. 2019; 9:1422–37. 10.1158/2159-8290.CD-18-1259 31340937 PMC7253691

[53] Zhang H, Zhang N, Dai Z, Wang Z, Zhang X, Liang X, Zhang L, Feng S, Wu W, Ye W, Zhang J, Luo P, Liu Z, et al. Hyaluronic acids mediate the infiltration, migration, and M2 polarization of macrophages: evaluating metabolic molecular phenotypes in gliomas. Mol Oncol. 2022; 16:3927–48. 10.1002/1878-0261.13315 36134697 PMC9718117

[54] Zhang H, Wang Y, Zhao Y, Liu T, Wang Z, Zhang N, Dai Z, Wu W, Cao H, Feng S, Zhang L, Cheng Q, Liu Z. PTX3 mediates the infiltration, migration, and inflammation-resolving-polarization of macrophages in glioblastoma. CNS Neurosci Ther. 2022; 28:1748–66. 10.1111/cns.13913 35855654 PMC9532932

[55] Ali HR, Chlon L, Pharoah PD, Markowetz F, Caldas C. Patterns of Immune Infiltration in Breast Cancer and Their Clinical Implications: A Gene-Expression-Based Retrospective Study. PLoS Med. 2016; 13:e1002194. 10.1371/journal.pmed.1002194 27959923 PMC5154505

[56] Bense RD, Sotiriou C, Piccart-Gebhart MJ, Haanen JB, van Vugt MA, de Vries EG, Schröder CP, Fehrmann RS. Relevance of Tumor-Infiltrating Immune Cell Composition and Functionality for Disease Outcome in Breast Cancer. J Natl Cancer Inst. 2016; 109:djw192. 10.1093/jnci/djw192 27737921 PMC6284248

[57] André P, Denis C, Soulas C, Bourbon-Caillet C, Lopez J, Arnoux T, Bléry M, Bonnafous C, Gauthier L, Morel A, Rossi B, Remark R, Breso V, et al. Anti-NKG2A mAb Is a Checkpoint Inhibitor that Promotes Anti-tumor Immunity by Unleashing Both T and NK Cells. Cell. 2018; 175:1731–43.e13. 10.1016/j.cell.2018.10.014 30503213 PMC6292840

[58] Joyce JA, Fearon DT. T cell exclusion, immune privilege, and the tumor microenvironment. Science. 2015; 348:74–80. 10.1126/science.aaa6204 25838376

[59] Queirolo P, Boutros A, Tanda E, Spagnolo F, Quaglino P. Immune-checkpoint inhibitors for the treatment of metastatic melanoma: a model of cancer immunotherapy. Semin Cancer Biol. 2019; 59:290–7. 10.1016/j.semcancer.2019.08.001 31430555

[60] Jiang P, Gu S, Pan D, Fu J, Sahu A, Hu X, Li Z, Traugh N, Bu X, Li B, Liu J, Freeman GJ, Brown MA, et al. Signatures of T cell dysfunction and exclusion predict cancer immunotherapy response. Nat Med. 2018; 24:1550–8. 10.1038/s41591-018-0136-1 30127393 PMC6487502

