# Supplementary material for: Identification of immune-related genes and integrated analysis of immune-cell infiltration in melanoma
Source: Aging (Albany NY). 2024 Jan 11;16(1):911–27. doi: 10.18632/aging.205427 (PMC10817386; doi:10.18632/aging.205427)
Supplement: Supplementary Material 1 [file aging-16-205427-s001.docx]

**Supplementary Material 1**

1. Abbreviation list

| RNA-sequencing | RNA-seq |
| --- | --- |
| Fragments Per Kilobase Million | FPKM |
| the Cancer Genome Atlas | TCGA |
| Least Absolute Shrinkage and Selection Operator | LASSO |
| risk score | RS |
| FC | Foldchange |
| ceRNA | competing endogenous RNA |
| Estimation of Stromal and Immune cells in malignant tumor tissues using Expression data | ESTIMATE |
| tumor microenvironment | TME |
| Tumor Immune Dysfunction and Exclusion | TIDE |
| tumor-associated macrophages | TAMs |
| myeloid-derived suppressor cells | MDSCs |
| cancer-associated fibroblasts | CAFs |
| Immune checkpoint inhibitor | ICI |

2. The datasets involved in this study

| GSE22153 | https://www.ncbi.nlm.nih.gov/geo/query/acc.cgi?acc=GSE22153 |
| --- | --- |
| GSE65904 | https://www.ncbi.nlm.nih.gov/geo/query/acc.cgi?acc=GSE65904 |
| GSE19234 | https://www.ncbi.nlm.nih.gov/geo/query/acc.cgi?acc=GSE19234 |
| mRNA of TCGA-SKCM | https://xenabrowser.net/datapages/?dataset=TCGA-SKCM.htseq_fpkm.tsv&host=https%3A%2F%2Fgdc.xenahubs.net&removeHub=https%3A%2F%2Fxena.treehouse.gi.ucsc.edu%3A443 |
| miRNA of TCGA-SKCM | https://xenabrowser.net/datapages/?dataset=TCGA-SKCM.mirna.tsv&host=https%3A%2F%2Fgdc.xenahubs.net&removeHub=https%3A%2F%2Fxena.treehouse.gi.ucsc.edu%3A443 |
| mutation of TCGA-SKCM | https://xenabrowser.net/datapages/?dataset=TCGA-SKCM.mutect2_snv.tsv&host=https%3A%2F%2Fgdc.xenahubs.net&removeHub=https%3A%2F%2Fxena.treehouse.gi.ucsc.edu%3A443 |

3. Code to filter out key immune-related genes

| rm(list=ls())  options(stringsAsFactors = F)  library(survival)  library(survminer)  dat=read.csv(file = 'dat.txt',sep = ',',header = T)  rownames(dat)=dat$ID  rt=dat[,-1]  rt=rt[,-1]  library(lars)  library(glmnet)  x=as.matrix(rt[,c(3:ncol(rt))])  y=rt$event  model_lasso <- glmnet(x, y, family="binomial", nlambda=50, alpha=1)  print(model_lasso)  head(coef(model_lasso, s=c(model_lasso$lambda[29],0.009)))  plot(model_lasso, xvar = "norm", label = TRUE)  plot(model_lasso, xvar="lambda", label=TRUE)  set.seed(2)  cv_fit <- cv.glmnet(x=x, y=y, alpha = 1, nlambda = 1000)  plot(cv_fit)  c(cv_fit$lambda.min,cv_fit$lambda.1se)  model_lasso <- glmnet(x=x, y=y, alpha = 1, lambda=cv_fit$lambda.1se)  lasso.prob <- predict(cv_fit, newx=x , s=c(cv_fit$lambda.min,cv_fit$lambda.1se) )  re=cbind(y ,lasso.prob)  dat=as.data.frame(re[,1:2])  colnames(dat)=c('event','prob')  dat$event=as.factor(dat$event)  library(ggpubr)  p <- ggboxplot(dat, x = "event", y = "prob",  color = "event", palette = "jco",  add = "jitter")  p + stat_compare_means()  library(ROCR)  library(glmnet)  library(caret)  pred <- prediction(re[,2], re[,1])  perf <- performance(pred,"tpr","fpr")  performance(pred,"auc") # shows calculated AUC for model  plot(perf,colorize=FALSE, col="black") # plot ROC curve  lines(c(0,1),c(0,1),col = "gray", lty = 4 )  fit <- glmnet(x=x, y=y, alpha = 1, lambda=cv_fit$lambda.1se)  head(fit$beta)  choose_gene=rownames(fit$beta)[as.numeric(fit$beta)!=0]  length(choose_gene)  myexpr=x[,choose_gene]  mysurv=rt[,c("time","event")]  mysurv$time=as.numeric(mysurv$time)  fit <- glmnet( myexpr, Surv(mysurv$time,mysurv$event),  family = "cox")  plot(fit, xvar="lambda", label = TRUE)  plot(fit, label = TRUE)  e=x[,choose_gene]  d=rt[,1:2]  dat=cbind(d,e)  colnames(dat)  s=Surv(time, event) ~ KIR2DL4+IFITM1+GPI+LIF+ADCYAP1R1+NTS  model <- coxph(s, data = dat )  summary(model,data=dat)  options(scipen=1)  ggforest(model, data =dat,  main = "Hazard ratio",  cpositions = c(0.10, 0.22, 0.4),  fontsize = 1.0,  refLabel = "1", noDigits = 4) |
| --- |
